# Ipsi- and contralateral frontal cortex oxygenation during handgrip task does not follow decrease on maximal force output

**DOI:** 10.1186/s40101-015-0077-z

**Published:** 2015-11-04

**Authors:** Naomi Kuboyama, Kenichi Shibuya

**Affiliations:** Faculty of Business Administration, Osaka University of Commerce, 4-1-10 Mikuriyasakae-machi, 577-8505 Higashi-Osaka, Japan; Department of Health and Nutrition, Niigata University of Health and Welfare, 1398 Shimami-chi, 950-3198 Niigata, Kita-Ku Japan

**Keywords:** Contralateral frontal cortex, Ipsilateral cortex, Maximal exercise, NIRS

## Abstract

The effect of fatiguing exercise on the ipsi- and contralateral frontal cortex has not been fully clarified. The purpose of this study was to investigate by near-infrared spectroscopy (NIRS) the frontal cortex oxygenation response to a prolonged fatiguing repetitive handgrip exercise performed at maximal voluntary contraction. It was found a significant oxyhemoglobin concentration ([HbO_2_]) increase (*p* < 0.05), accompanied by a smaller and delayed deoxyhemoglobin concentration ([Hb]) decrease (*p* < 0.05), in both hemispheres. Then, it was indicated higher delayed oxygenation in ipsilateral oxygenation compared to contralateral oxygenation. These results provide further evidence that the complemental interaction between the ipsilateral and contralateral cortex during the fatiguing maximal exercise.

## Introduction

Muscle fatigue induces progressive changes in muscle and brain. During fatiguing exercise, muscle fibers contract repeatedly, depleting energy supplies, and eventually resulting in muscle fatigue. Muscle fatigue is defined as “an exercise-induced loss of the power- and force-generating ability of muscle during or after exercise” [4, 18].

Several studies have examined the factors responsible for muscle fatigue that determine the limits of exercise capacity. Numerous investigators have focused on the peripheral factors of muscle fatigue [14, 16]. However, the influence of central fatigue on exercise capacity has received little attention. Recently, the development of NIRS has made it possible to determine cerebral cortex activity during exercise [5, 15, 22]. Several of these NIRS studies have reported that cerebral cortex activity decreases at the point of voluntary exhaustion during fatiguing exercises [3, 25, 26].

These studies examined the changes in cerebral oxygenation during submaximal contraction [21, 22]. While muscle fatigue induces progressive changes in muscle and brain, it is unknown in submaximal contraction when muscle fatigue develops. Nor have previous studies compared cerebral cortex oxygenation and muscle activity (electromyography (EMG) activity) [20, 21]. To fully understand muscle fatigue, it is necessary to investigate cerebral cortex activity during voluntary maximal contraction, as well as the relationship between muscle activity and cerebral cortex activity.

NIRS has recently attracted the interest of researchers using brain-machine interfaces (BMIs) [7]. Unlike electroencephalography (EEG), NIRS is not corrupted by electromagnetic noise. Also, it is portable, unlike functional magnetic resonance imaging (fMRI). These recent studies have revealed the influence of skin blood flow on NIRS signals in humans [11, 12]. It is very important to understand the effect of fatiguing skeletal muscle exercise on the brain—in particular on the ipsi- and contralateral frontal cortex—in order to apply NIRS data to BMIs. If it was possible to observe the difference between the ipsilateral and contralateral frontal cortex oxygenation, or even if there were synchronization between NIRS oxygenation and skin blood flow, it would be possible to know the activation pattern of the ipsi- and contralateral frontal cortex.

In this study, we investigated NIRS signals on the ipsi- and contralateral frontal cortex during repetitive voluntary maximal exercise. Our hypothesis was that the oxygenation of the ipsilateral frontal cortex might be delayed compared with that of the contralateral cortex for complementary function of the two. This would be consistent with previous research focused on the primary motor cortex oxygenation during submaximal exhaustive exercise [22].

## Methods

### Subjects

Eight right-handed males participated in the study. Their mean (±SE) age, height, and weight were 23 years (± 0.7), 173 cm (±1.1), and 70 kg (±2.8), respectively. Subjects were asked to lie in a supine position on a mat and perform a maximal handgrip task (3-s contractions/3-s rest, 50 cycles).

Subjects adjusted the handgrip force of the instrument before beginning the study. Handgrip force was measured with a system consisting of a handgrip device. The sampling rate for force data was 33 Hz. Subjects exerted handgrip contractions as they listened to a digital sound generated by the sound-stimuli software. When the digital sound was beeping (3 s), the subjects were asked to exert maximum force and maintain it until the sound stopped. When there was no digital sound (3 s), subjects were asked to relax and not exert any force. Subjects repeated this process 50 times.

### Near-infrared spectroscopy

Blood oxygenation level-dependent evaluations of cerebral blood flow (CBF) are important to imaging studies of brain function [27], whereas the blood oxygen level-dependent (BOLD) control of brain oxygen diffusion is the basis for an emerging understanding of the neuroenergetics of brain function [8]. Capillary oxygenation can be monitored noninvasively by NIRS in real time during situations ranging from surgery [28] to strenuous whole body exercise [17]. We used a three-wavelength (775, 810, and 850 nm) NIRS (NIRO-200; Hamamatsu Photonics, Japan) to measure ipsi- and contralateral frontal cortex oxygenation. The NIRO-200 makes it possible to quantify tissue oxygenation. However, this measurement requires optically homogenous tissue so that the signal-to-noise ratio can be reduced, and this is unlikely to be strictly the case over cerebral tissue [10]. As the accuracy of this parameter is under discussion, at least concerning cerebral tissue, we used cerebral [Hb_diff_] values to evaluate cerebral activity in this study.

The optical probe was comprised of one emitter and one detector (with a total of three separate sensors), guided onto subjects’ heads through glass fiber bundles. The probes were positioned over the bilateral frontal cortex areas (AF3 and AF4) according to the modified international EEG 10–20 system. The distance between the transmitting and receiving probes was 4.0 cm. The probes positioned around the ipsi- and contralateral frontal area were checked by a right-hand grip task to induce functional oxygenation. If no oxygenation changes were detected in response to the handgrip task, the probes were moved by several millimeter until a consistent oxygenation response was achieved by trial and error.

The probes were fixed to the ipsi- and contralateral frontal cortex areas with a light sealing tape and a strap. The NIRS data were collected with a sample frequency of 2 Hz. Relative concentration changes were measured from resting baseline of oxyhemoglobin ([HbO_2_]), deoxyhemoglobin ([Hb]), and hemoglobin difference ([Hb_diff_] = [HbO_2_] − [Hb]). [Hb] is known to be a reliable estimator of changes in tissue oxygenation status, while [HbO_2_] seems to be the most sensitive indicator of regional cerebral blood flow modifications [13]. [Hb_diff_] is known to be a good indicator of oxygenation [29, 31] due to its high correlation with cerebral blood flow [30]. To avoid inclusion of the effects of the preparation and/or attention to the exercise on the oxygenation changes in the analyses, we determined the baseline values of [HbO_2_], [Hb], and [Hb_diff_] as the mean value over 30 s before the onset of the contraction phase. Since the baseline was stable, the [HbO_2_], [Hb], and [Hb_diff_] baselines were expressed as zero, and [HbO_2_], [Hb], and [Hb_diff_] parameters are presented as the magnitude of change from the respective baseline. The values of [HbO_2_], [Hb], and [Hb_diff_] during exercise were calculated as an average of six data points (3 s) during each contraction.

### EMG activity

Surface EMGs from the muscle bellies of the right finger flexor muscles were recorded by means of bipolar Ag/AgCl electrodes with an inter-electrode distance of 20 mm. Low impedance between the two bipolar electrodes (<5 kΩ) was obtained by shaving, abrading and washing the skin with emery paper, and then cleaning it with 90 % alcohol. The reference electrode was placed on the elbow of the same arm. In order to minimize movement artifacts, electrodes and cables were strapped onto subjects using an elastic muff net. EMG activity was recorded continuously from warm-up to the end of exercise via a dedicated acquisition system. The EMG signals were amplified (×1000), band-pass filtered (30–500 Hz), and sampled at 1000 Hz. They were then integrated (iEMG) for the subsequent analyses. The peak iEMG for handgrip was determined from an interval between the start of the contraction and a point 0.5 s beyond the peak level of the force curve. The values of iEMG during exercise were calculated as an average of 3 s during each contraction.

### Statistical analyses

The average values were expressed as mean (SE). The criterion for significance was *p* < 0.05. In order to determine the significance of [HbO_2_], [Hb], [Hb_diff_], iEMG, and force changes, one-way repeated measure analysis of variance (ANOVA) and post-hoc Dunnet test were performed. To assess the difference in oxygenation kinetics between hemispheres, it was compared with the timing of the peak value in [Hb_diff_], and then, Student’s paired *t* test was performed.

## Results

### Contralateral frontal cortex oxygenation changes during maximal handgrip task

**Fig. 1:**
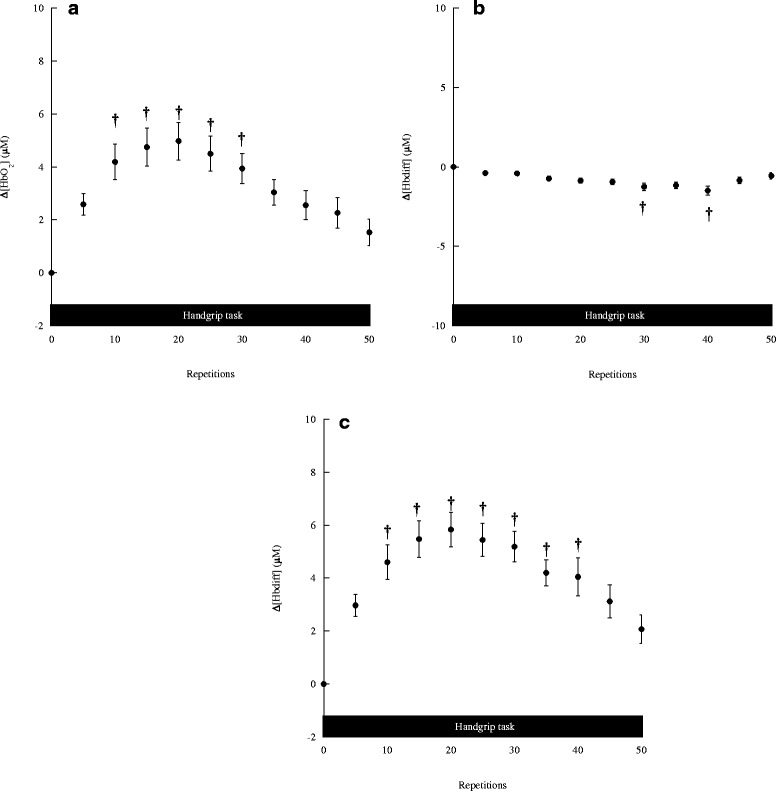
The (**a**) [HbO_2_], (**b**) [Hb], and (**c**) [Hb_diff_] changes in the contralateral frontal cortex during handgrip task. Values are mean ± SE. Asterisks indicate significant differences compared to the baseline values, *p* < 0.05

All subjects completed the designated maximal handgrip task. The changes in [HbO_2_], [Hb] and [Hb_diff_] in the contralateral frontal cortex during the motor task are shown in Fig. 1, respectively. The contralateral frontal [HbO_2_] levels changed significantly during the motor task (*F* (10, 70) = 2.844, *p* < 0.01) (Fig. 1a). Compared with the baseline value, the contralateral frontal [HbO_2_] levels markedly increased from trial 10–30 trials (*p* < 0.05); they then gradually decreased from trial 35 on. The contralateral frontal [Hb] levels also changed significantly during the motor task (*F* (10, 70) = 2.068, *p* < 0.05) (Fig. 1b). Compared with the baseline value, the contralateral frontal [Hb] levels were decreased at 30 trials and 40 trials (*p* < 0.05). The contralateral frontal [Hb] gradually increased after 40 trials. Meanwhile, the contralateral frontal [Hb_diff_] levels significantly increased during the motor task (*F* (10, 70) = 3.347, *p* < 0.005) (Fig. 1c). The contralateral frontal [Hb_diff_] significantly increased from trial 10–40; it then gradually decreased after 45 trials.

### Ipsilateral frontal cortex oxygenation changes during maximal handgrip task

**Fig. 2:**
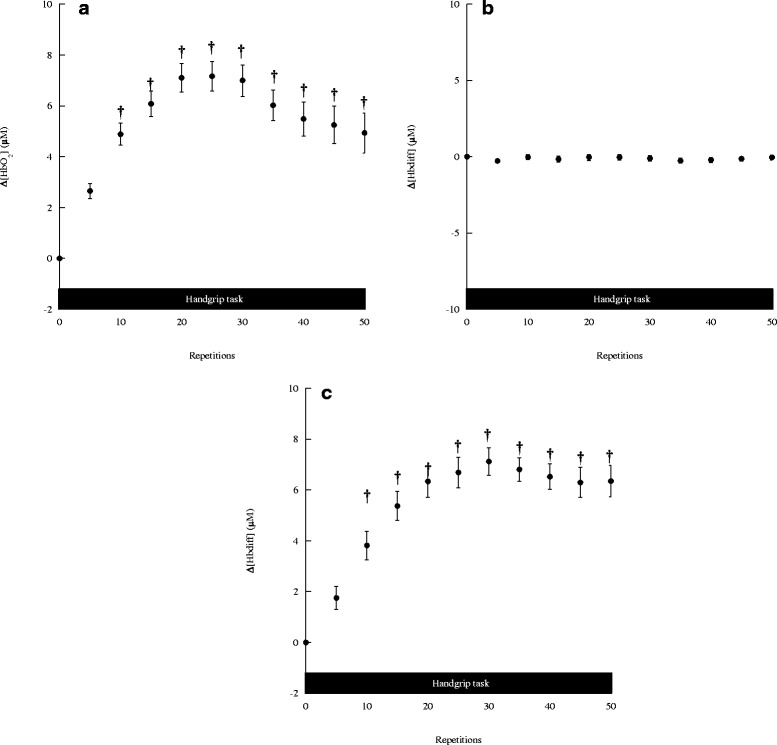
The (**a**) [HbO_2_], (**b**) [Hb], and (**c**) [Hb_diff_] changes in the ipsi-lateral frontal cortex during handgrip task. Values are mean ± SE. Asterisks indicate significant differences compared to the baseline values, *p* < 0.05

The changes in [HbO_2_], [Hb] and [Hb_diff_] in the ipsilateral frontal cortex during the motor task are shown in Fig. 2, respectively. The [HbO_2_] levels in the ipsilateral frontal cortex changed significantly during the motor task (*F* (10, 70) = 3.934, *p* < 0.001) (Fig. 2a). Compared with the baseline value, the ipsilateral frontal [HbO_2_] levels markedly increased from trials 5–50 (*p* < 0.05). Meanwhile, the ipsilateral frontal [Hb] levels did not change significantly during the motor task (*F* (10, 70) = 0.180, *p* > 0.05) (Fig. 2b). The ipsilateral frontal [Hb_diff_] levels significantly increased during the motor task (*F* (10, 70) = 5.584, *p* < 0.001) (Fig. 2c). Ipsilateral frontal [Hb_diff_] significantly increased from trial 15–50.

#### The kinetic analysis in bilateral M1 oxygenation

To assess the difference of oxygenation kinetics between the ipsi- and contralateral cortex, it was compared with the timing of the peak values in [Hb_diff_] after the start of exercise. The average time of timing of the peak value in [Hb_diff_] of the contralateral cortex was 18.12 (2.4) s after the start of exercise. On the other hand, the average time of timing of the peak value in [Hb_diff_] of the contralateral cortex was 33.75 (5.5) s after the start of exercise. The oxygenation kinetics during exercise were significantly faster in the contralateral cortex than the ipsilateral cortex (*t* = −2.5384, *p* < 0.05).

### Force output and iEMG changes during exercise

**Fig. 3:**
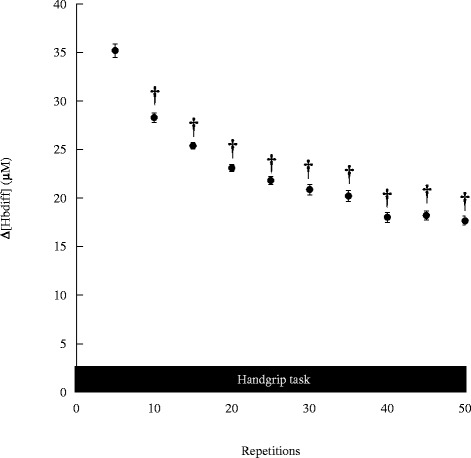
Force changes during handgrip task. Values are mean ± SE. Asterisks indicate significant differences compared to the baseline values, *p* < 0.05

**Fig. 4:**
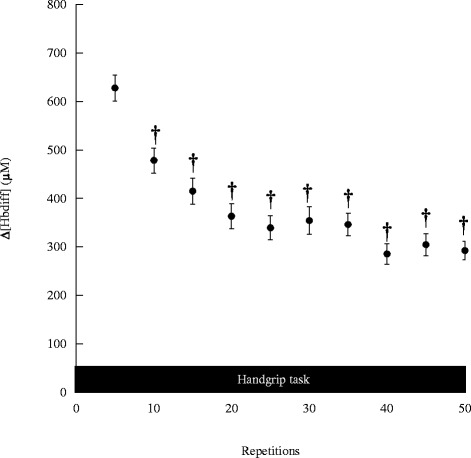
iEMG changes during handgrip task. Values are mean ± SE. Asterisks indicate significant differences compared to the baseline values, *p* < 0.05

The corresponding force output kinetics during the motor task are shown in Fig. 3. The force levels changed significantly during the motor task (*F* (10, 70) = 31.326, *p* < 0.001). Compared with the values at the first 5 trials, the force levels significantly decreased after 10 trials (*p* < 0.001). The iEMG kinetics during the motor task is shown in Fig. 4. The iEMG levels changed significantly during the motor task (*F* (10, 70) = 51.793, *p* < 0.001). Compared with the value during the first 5 trials, iEMG levels significantly decreased after 10 trials (*p* < 0.001).

## Discussion

Changes in cerebral oxygenation reflect cerebral functional activation [5, 15, 19]. In the present study, we examined the interaction of ipsi- and contralateral frontal cortex activity and the force of MVC contraction during a repetitive maximal handgrip task using NIRS. The main results were as follows: (1) that contralateral frontal cortex activity does not follow the changes in the force of MVCs throughout a repetitive MVC handgrip task, (2) that the activity of the ipsilateral frontal cortex increases throughout the task, (3) that the activity of the ipsilateral frontal cortex also does not follow the changes of the force of MVCs, and (4) that the activity in the contralateral frontal cortex increased after the start of the exercise and then gradually decreased to the level of the resting values.

We found a strong relationship between the changes in the force and the iEMG during a repetitive MVC handgrip task. The motor command would be sent from the supraspinal region during the motor task. We found different kinetics in the contralateral and ipsilateral frontal cortex during the motor task. The activity of the contralateral frontal cortex increased and then decreased during the motor task, while that of the ipsilateral frontal cortex increased throughout the motor task.

One previous study suggested a connection between the ipsilateral and contralateral cortex during the handgrip task [2]. Shibuya and Kuboyama [22] also reported the possibility of connection between the ipsilateral and contralateral cortex during a submaximal fatiguing exercise. In another study, Shibuya et al. [24] suggested the ipsilateral cerebral cortex provided complementary activity to the insufficient activity of the contralateral cerebral cortex. The results of the present study would also suggest insufficient activity in the contralateral frontal cortex to the changes in the force output. To be complementary, the activity in the ipsilateral frontal cortex would keep increasing throughout the motor task. The results also suggested that the connection between the bilateral cerebral cortex would occur during exercise, not before it began [24]. In addition, previous studies [1, 23] reported that the supraspinal fatiguing proposed by Gandevia [9] would appear in the subcortical region in normal subjects. However, in the normal subjects of the current study, the activity in the contralateral cerebral cortex did not cause an increase of the force output during the motor task. Durduran et al. [6] reported the artifacts on NIRS signals induced by changes to head inclination. To avoid the artifacts induced by the changes to head inclination, we evaluated the oxygenation by [Hb_diff_]. [Hb_diff_] is known to be a good indicator of oxygenation [29, 31] due to its high correlation with cerebral blood flow [30].

In conclusion, the results of the present study show different oxygenation kinetics between the ipsilateral and contralateral frontal cortex during the course of a motor task. The increasing oxygenation in the ipsilateral frontal cortex suggests a complementary interaction between both hemispheres during such a task.

In conclusion, it was found a significant [HbO_2_] increase, accompanied by a smaller and delayed [Hb] decrease, in both hemispheres. Then, it was indicated higher delayed oxygenation in ipsilateral oxygenation compared to contralateral oxygenation. These results provide further evidence of the complemental interaction between the ipsilateral and contralateral cortex during the fatiguing maximal exercise.
